# Shear wave elastography to unmask differences in myocardial stiffness between athletes and sedentary non-athletes

**DOI:** 10.1093/ehjimp/qyaf023

**Published:** 2025-03-21

**Authors:** Karim Taha, Youri Bekhuis, Ruben de Bosscher, Christophe Dausin, Marta Orlowska, Ahmed S Youssef, Stéphanie Bézy, Véronique Cornelissen, Lieven Herbots, Rik Willems, Jens-Uwe Voigt, Jan D’hooge, Guido Claessen

**Affiliations:** Division of Heart and Lungs, Department of Cardiology, University Medical Center Utrecht, Heidelberglaan 100, P.O. Box 85500, 3508 GA, Utrecht, The Netherlands; Department of Cardiovascular Sciences, KU Leuven, Herestraat 49, 3000 Leuven, Belgium; Division of Cardiology, University Hospitals Leuven, Herestraat 49, 3000 Leuven, Belgium; Department of Cardiovascular Sciences, KU Leuven, Herestraat 49, 3000 Leuven, Belgium; Division of Cardiology, University Hospitals Leuven, Herestraat 49, 3000 Leuven, Belgium; Department of Cardiovascular Sciences, KU Leuven, Herestraat 49, 3000 Leuven, Belgium; Division of Cardiology, University Hospitals Leuven, Herestraat 49, 3000 Leuven, Belgium; Department of Movement Sciences, KU Leuven, Leuven, Belgium; Department of Cardiovascular Sciences, KU Leuven, Herestraat 49, 3000 Leuven, Belgium; Department of Cardiovascular Sciences, KU Leuven, Herestraat 49, 3000 Leuven, Belgium; Department of Cardiovascular Medicine, Suez Canal University, Ismailia, Egypt; Department of Cardiovascular Sciences, KU Leuven, Herestraat 49, 3000 Leuven, Belgium; Research Group of Rehabilitation of Internal Disorders, Department of Rehabilitation Sciences, Faculty of Movement and Rehabilitation Sciences, KU Leuven, Leuven, Belgium; Department of Medicine and Life Sciences, University of Hasselt, Hasselt, Belgium; Division of Cardiology, Hartcentrum, Jessa Ziekenhuis, Hasselt, Belgium; Department of Cardiovascular Sciences, KU Leuven, Herestraat 49, 3000 Leuven, Belgium; Division of Cardiology, University Hospitals Leuven, Herestraat 49, 3000 Leuven, Belgium; Department of Cardiovascular Sciences, KU Leuven, Herestraat 49, 3000 Leuven, Belgium; Division of Cardiology, University Hospitals Leuven, Herestraat 49, 3000 Leuven, Belgium; Department of Cardiovascular Sciences, KU Leuven, Herestraat 49, 3000 Leuven, Belgium; Department of Cardiovascular Sciences, KU Leuven, Herestraat 49, 3000 Leuven, Belgium; Department of Medicine and Life Sciences, University of Hasselt, Hasselt, Belgium; Division of Cardiology, Hartcentrum, Jessa Ziekenhuis, Hasselt, Belgium

**Keywords:** shear wave elastography, high frame rate imaging, stress echocardiography, myocardial stiffness, diastolic function

## Abstract

**Aims:**

Myocardial stiffening naturally occurs with aging and contributes to diastolic dysfunction. Assessing myocardial stiffness non-invasively could improve the sensitivity of diastolic function evaluation in clinical practice. Shear wave (SW) elastography is a non-invasive tool for quantifying myocardial stiffness, where higher SW velocities indicate increased stiffness. We investigated whether SW elastography could detect differences in myocardial stiffness between athletes and sedentary non-athletes and, during exercise, reveal differences in operational stiffness that may indicate diastolic dysfunction.

**Methods and results:**

We enrolled 20 master athletes (median age 60 [IQR 59–66] years) and 17 sedentary non-athletes (median age 58 [IQR 52–71] years). Standard exercise echocardiography revealed no significant differences in diastolic function between the groups. Additionally, ultra-high frame rate imaging was used to measure SW velocities after mitral valve closure (MVC) and aortic valve closure (AVC) at rest and during exercise. At rest, athletes had lower SW velocities after MVC compared to sedentary non-athletes (3.2 ± 0.4 m/s vs. 3.9 ± 0.7 m/s, respectively, *P* = 0.003). During exercise, SW velocities after AVC significantly increased in sedentary non-athletes but not in athletes (+1.6 ± 1.6 cm/s increase per 1% power output increase vs. 0.0 ± 0.8 cm/s, respectively, *P* = 0.006). An inverse correlation was found between the increase of SW velocity after AVC during exercise and VO_2_max (*r* = −0.51, *P* = 0.003).

**Conclusion:**

SW elastography reveals reduced myocardial stiffness in athletes compared to sedentary non-athletes at rest and during exercise, which is not detected by conventional echocardiographic measurements. Exercise-induced changes in SW velocities after AVC may potentially serve as an early marker for detecting diastolic dysfunction.

## Graphical Abstract

**Graphical Abstract:**
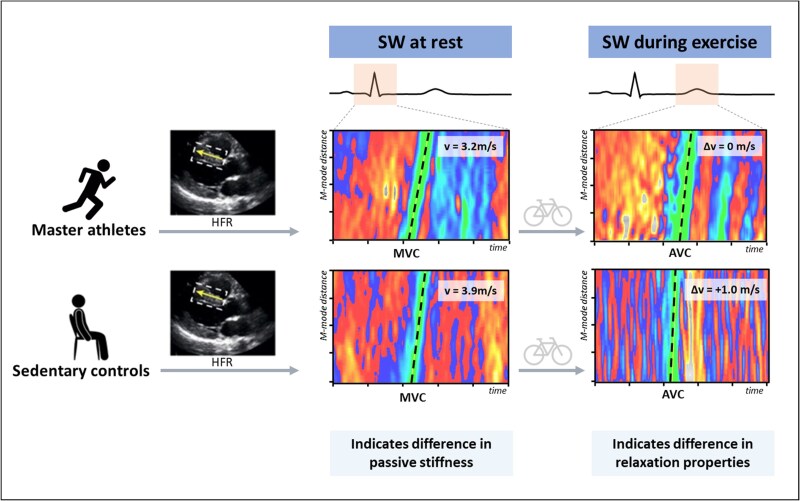
Shear wave (SW) elastography unmasks stiffness differences at rest and during exercise between athletes and sedentary controls. Ultra-high frame rate imaging was performed at rest and during exercise in athletes and controls. M-mode maps, showing acceleration of tissue, were extracted from the midline of the interventricular septum. The SWs after mitral valve closure (MVC) and aortic valve closure (AVC) appear as tilted bands on the acceleration maps, where steeper bands represent higher velocities. The presented velocities represent the mean velocities in the groups. At rest, SW velocity after MVC revealed reduced passive stiffness in athletes, whereas during exercise, SW velocity after AVC revealed reduced operational stiffness during isovolumic relaxation in athletes. AVC, aortic valve closure; HFR, high frame rate; IVRT, isovolumic relaxation time; MVC, mitral valve closure; SW, shear wave; v, velocity.

## Introduction

Cardiovascular stiffening is a process that naturally occurs with aging.^1–3^ This process can be accelerated by certain risk factors, such as hypertension, obesity, insulin resistance, and a sedentary lifestyle. Importantly, stiffening of the myocardium leads to decreased compliance of the left ventricle and impaired diastolic performance, which may eventually progress to clinical heart failure.^4,5^ Nowadays, heart failure due to diastolic dysfunction is recognized as an important cause of heart failure; approximately half of the patients with heart failure are found to have heart failure with preserved ejection fraction.^6,7^

The non-invasive assessment of diastolic function by echocardiography remains challenging. The use of exercise echocardiography is emerging, since imaging during exercise has been shown to provide additional insight into diastolic function.^8,9^ However, most of the currently measured parameters reflect advanced diastolic dysfunction, such as increased filling pressures and pulmonary hypertension, but none of the currently available parameters directly reflects intrinsic stiffness of the myocardium.^10^ This hampers the sensitivity of diastolic function assessment. Yet, the detection of earlier signs of diastolic dysfunction is of great clinical interest since early initiation of lifestyle interventions and therapeutic interventions has potential to slow down the progression of diastolic dysfunction and prevent clinical heart failure.^11,12^

Shear wave (SW) elastography is a non-invasive tool that allows for quantification of myocardial tissue properties.^13,14^ SWs are mechanical waves propagating through the myocardium that can be induced artificially by an external ultrasound impulse.^15^ Besides external impulses, SWs are also induced naturally by physiological events such as the closure of the mitral valve or the aortic valve.^16^ The speed of SW propagation depends directly on the extent of myocardial stiffness: higher SW propagation velocities indicate increased myocardial stiffness. Since SWs are short-lived events that attenuate quickly, they can only be captured by ultra-high frame rate (HFR) imaging.^17^ Several clinical applications of SW elastography have been investigated previously.^15,18–20^ The non-invasive assessment of myocardial stiffness by SW elastography has great potential to push the frontiers of diastolic function assessment in clinical practice, possibly allowing for identification of earlier stages of diastolic dysfunction.

Prior invasive studies have demonstrated athletes to have reduced chamber stiffness and enhanced left ventricular (LV) compliance in comparison to sedentary control subjects.^3,21^ The present study aims to explore the potential of SW elastography to detect these differences non-invasively. The two main objectives are as follows: (i) to investigate whether SW elastography can reveal differences in intrinsic myocardial stiffness between athletes and sedentary non-athletes and (ii) to study whether SW elastography can be used during exercise to detect differences in operational stiffness as a potential indicator of diastolic dysfunction.

## Methods

### Study population

This study was performed at the University Hospital Leuven in Leuven, Belgium, between May 2021 and May 2022. We recruited master endurance athletes from the Master@Heart study.^22^ These athletes were males aged 45–70 years and had been engaged in long-term intensive endurance sports: ≥8 h per week for cyclists and ≥6 h per week for runners. Besides the athletes, we enrolled non-athletic male control subjects who have not been involved in regular sports practice ≥3 h per week. These control subjects were enrolled both from the Master@Heart study^22^ and from an ongoing randomized trial investigating preventive exercise therapy in sedentary subjects.^23^ Patients with cardiomyopathies, coronary artery disease, or any signs of structural or functional abnormalities, including diastolic dysfunction, were excluded.^10,24^

### Study design

First, the study participants underwent standard cardiopulmonary exercise testing (CPET) on a bicycle to determine the maximum oxygen uptake (VO_2_max) and the maximum exercise power.^22^ Next, we performed bicycle exercise echocardiography on a programmable, electronically braked, semi-supine ergometer (ergoline GmbH, Bitz, Germany). After acquiring both conventional images and ultra-HFR images at rest, the power output was gradually increased, typically in steps of 10 or 20 W per minute. Heart rate was monitored continuously, and blood pressure was measured during the image acquisition stages. Image acquisition was performed at 25, 50, and 75% of the maximum power output that was reached during baseline CPET. During image acquisition, the stepwise protocol was paused to keep the power output constant.

### Image acquisition

Exercise echocardiography was performed by one operator (K.T.) using a Vivid E95 machine equipped for ultra-HFR imaging (GE Healthcare, Horten, Norway). The full acquisition protocol is provided in Supplementary data online, *Table S1*. The conventional images were all acquired using a standard M3S phased array probe. To enhance the tricuspid regurgitation (TR) signal for estimation of pulmonary artery pressures, agitated colloid was infused intravenously.^25^ In addition to the conventional images, HFR recordings were acquired with a phased array probe that allows for diverging transmitted waves, leading to fast image sequences exceeding 1000 frames per second. Using the HFR mode, three parasternal long-axis views were acquired per stage, with the aortic valve and mitral valve clearly visible to enable the identification of event timings.

### Image analysis and SW measurements

Conventional images were analysed offline according to standard recommendations using EchoPAC version 204 (GE Healthcare, Horten, Norway).^24^

The method for SW velocity measurement was previously described in detail.^18–20^ In brief, an in-house developed software was used for post-processing of the HFR data (SPEQLE version 4.6.8, KU Leuven). Tissue acceleration was calculated from the tissue Doppler velocity estimates from the HFR data. Acceleration M-mode maps were extracted from the midline of the interventricular septum (*Graphical Abstract*). The SWs of interest, appearing on the M-mode as tilted colour bands, were identified after MVC and AVC. The slopes of the SWs were measured semiautomatically. Steeper slopes represent higher velocities. Per exercise stage, the measurements were performed on three separate beats and the results were averaged.

### Statistical analysis

Data are provided as mean ± standard deviations or as median [interquartile ranges], as appropriate. Values were compared between groups using an independent samples *t*-test or a Mann–Whitney *U* test as appropriate. Values between different exercise stages within a group were compared using a paired *t*-test or a Wilcoxon signed-rank test as appropriate. Proportions were compared between groups using a *χ*^2^ test or Fisher’s exact test as appropriate. For every individual participant, a linear regression line for the consecutive SW velocities during exercise was calculated for the SW velocities after MVC and AVC. The slopes of these regression lines reflect the change in SW velocity per 1% increase in power output and were compared between athletes and non-athletes. Linear correlations between two variables were measured using the Pearson correlation coefficient. To measure intra- and interobserver reproducibility, the SW measurements were blindly repeated in a random subset of 10 subjects by the first operator (K.T.) and by a second operator (A.S.Y). These measurements were used to calculate intraclass correlation coefficients (ICC). Two-sided *P* < 0.05 was considered to indicate statistical significance. Statistical analyses were performed with R Studio Version 1.3.1093 (R Foundation for Statistical Computing, Vienna, Australia) and IBM SPSS Statistics Version 29 (IBM Corporation, Armonk, NY).

## Results

### Study population and baseline measurements

Baseline characteristics of the study population are provided in *Table 1*. The study cohort consisted of 20 male athletes and 17 male control subjects who had comparable age and sex distribution (60 [59–66] years vs. 58 [52–71] years, respectively). While baseline systolic and diastolic blood pressure were equal between the groups, baseline heart rate was lower in the athletes than in the non-athletes (60 ± 9 bpm vs. 69 ± 7 bpm, respectively, *P* = 0.003). One control subject had a history of hypertension, but he did not show any signs of structural or functional cardiac abnormalities, including normal diastolic measurements.

**Table 1:** Baseline characteristics

	Master athletes (*n* = 20)	Non-athletes (*n* = 17)	*P*-value
Age (years)	60 [59–66]	58 [52–71]	0.259
Male sex	20 (100)	17 (100)	
Systolic BP (mmHg)	129 ± 12	136 ± 21	0.333
Diastolic BP (mmHg)	80 ± 12	80 ± 12	0.983
Heart rate (bpm)	60 ± 9	69 ± 7	0.003
VO_2_max (mL/min/kg)	47 ± 6	30 ± 7	<0.001
Echocardiography (rest)			
IVSd (mm)	9.1 ± 1.1	8.8 ± 1.6	0.026
LVPWd (mm)	9.3 ± 1.1	9.1 ± 1.4	0.121
LVEDV (mL)	141 ± 23	113 ± 19	<0.001
LVESV (mL)	67 ± 11	48 ± 11	<0.001
LVEF (%)	53 ± 4	58 ± 4	0.004
LV CO (mL)	5.3 ± 1.1	5.5 ± 1.0	0.672
LAVI (mL/m^2^)	39 ± 6	27 ± 6	0.001
LA reservoir strain (%)	30 ± 4	27 ± 8	0.225
E-wave velocity (cm/s)	65 ± 13	64 ± 10	0.757
E-wave DT (ms)	183 ± 65	199 ± 38	0.432
A-wave velocity (cm^2^/m^2^)	60 ± 13	64 ± 12	0.401
Average e′ (cm/s)	10 ± 1	10 ± 2	0.447
E/e′ ratio	8 ± 2	8 ± 2	0.772
TR max pressure gradient (mmHg)	20 ± 4	22 ± 4	0.151
SW velocities (rest)			
SW velocity MVC (m/s)	3.2 ± 0.4	3.9 ± 0.7	0.003
SW velocity AVC (m/s)	3.6 ± 0.5	3.6 ± 0.5	0.876

Values are presented as mean ± standard deviation or median [interquartile range]; *P* < 0.05 is considered to indicate statistical significance.

AVC, aortic valve closure; BP, blood pressure; BSA, body surface area; CO, cardiac output; DT, deceleration time; IVSd, interventricular septal thickness; LAVI, left atrial volume index; LV, left ventricular; LVEDV/LVESV, left ventricular end diastolic/systolic volume; LVEF, left ventricular ejection fraction; LVPWd, left ventricular posterior wall thickness; MVC, mitral valve closure; SW, shear wave; TR, tricuspid regurgitation.

Echocardiographic measurements are shown in *Table 1*. Athletes had higher interventricular septal thickness than control subjects, higher LV and left atrial volumes, and lower LVEF (53 ± 4% vs. 58 ± 4%, respectively, *P* = 0.004). None of the standard functional diastolic measurements at rest were significantly different between athletes and non-athletes.

At rest, SW velocity after MVC was lower in athletes than in non-athletes (3.2 ± 0.4 m/s vs. 3.9 ± 0.7 m/s, respectively, *P* = 0.003). Importantly, during the resting measurements, there was no correlation between SW velocity after MVC and heart rate (*r* = 0.093, *P* = 0.651). SW velocity after AVC was equal between athletes and non-athletes at rest (3.6 ± 0.5 m/s vs. 3.6 ± 0.5 m/s, respectively, *P* = 0.876).

### Exercise measurements

#### CPET and haemodynamics

CPET showed athletes to reach a higher VO_2_max than non-athletes (47 ± 6 mL/min/kg vs. 30 ± 7 mL/min/kg, respectively, *P* < 0.001).

The haemodynamic measurements during exercise echocardiography are shown in Supplementary data online, *Table S2*. During the consecutive exercise stages, athletes reached heart rates of 95 ± 12, 119 ± 14, and 149 ± 20 bpm. This was similar in non-athletes, who reached heart rates of 99 ± 8, 120 ± 11, and 147 ± 18 bpm (*P* > 0.05 for all). Systolic and diastolic blood pressure measurements during exercise were also similar among the two groups. Athletes reached a higher cardiac output than non-athletes during peak exercise (14.5 ± 2.7 L/min vs. 11.9 ± 2.0 L/min, respectively, *P* = 0.035).

#### Standard exercise echocardiography

**Figure 1:**
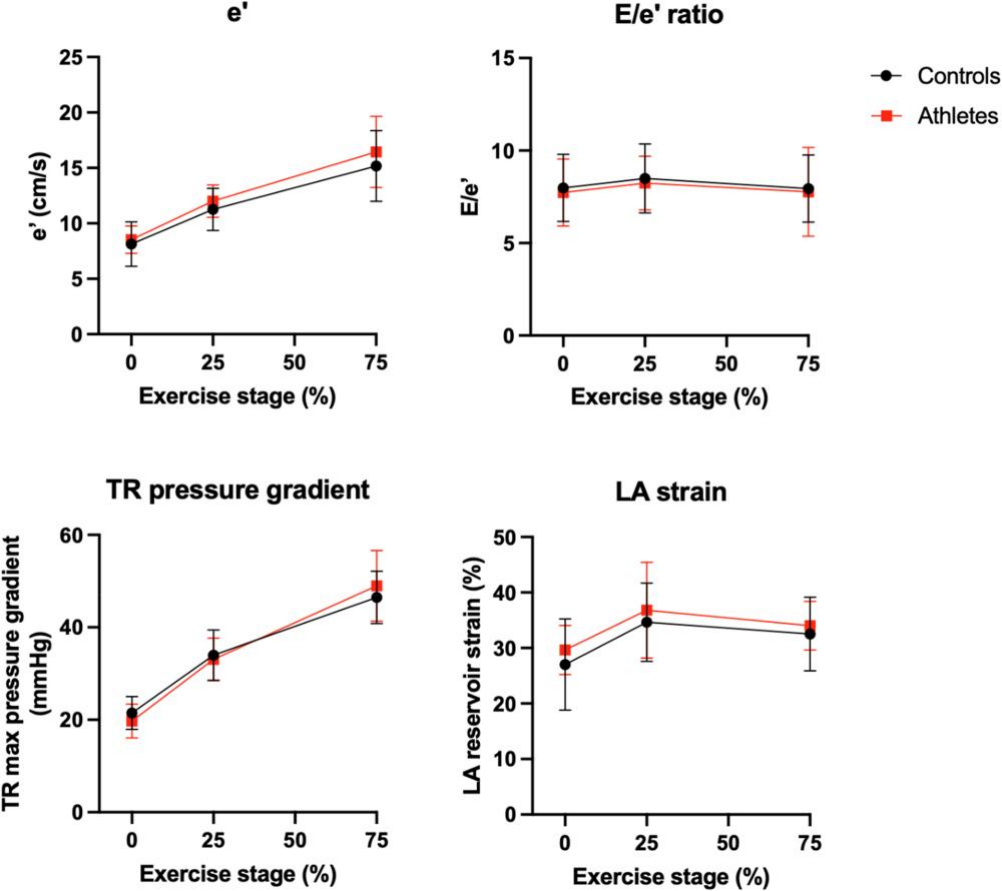
Evolution of conventional diastolic parameters during exercise. Conventional echocardiographic parameters were measured at rest and during exercise at 25 and 75% of maximal power output. The plotted values represent means and standard deviations. LA, left atrial; TR, tricuspid regurgitation.

The standard diastolic echocardiographic measurements during exercise are demonstrated in *Figure 1*. None of the parameters showed a significantly different delta between athletes and non-athletes.

#### Exercise SW elastography

**Figure 2:**
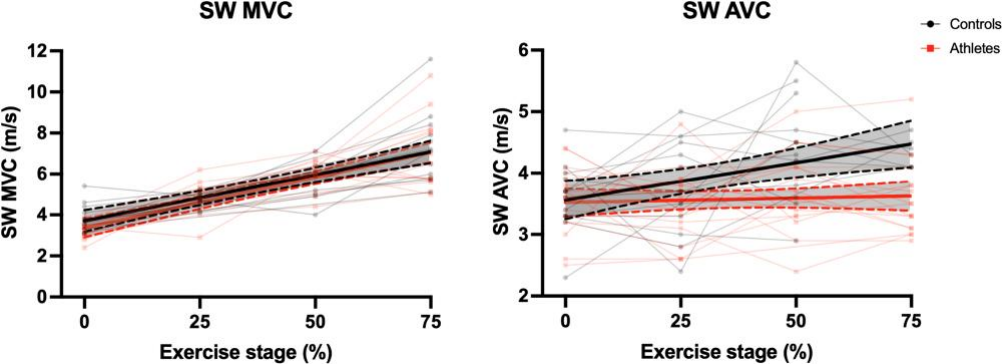
Evolution of SW velocities during exercise. The black and red lines show the mean linear regression lines for the athletes and non-athletes with the dotted lines representing the 95% CIs, for the SW velocity measurements after MVC (left) and after AVC (right). The individual SW velocity measurements for all individuals are plotted as well. SW AVC, shear wave velocity after aortic valve closure; SW MVC, shear wave velocity after mitral valve closure.

The SW velocities during exercise are demonstrated in *Figure 2*. The increase in SW velocities after MVC showed a linear trend in both groups. These velocities increased to similar values in both groups, and the linear slopes of these velocities were not significantly different between the groups (increase in SW velocity per 1% power output increase: 5.2 ± 2.0 cm/s in athletes vs. 4.4 ± 2.8 cm/s in non-athletes, *P* = 0.415). A similar trend was seen for the slopes of SW velocity change per heart rate increase (see Supplementary data online, *Figure S1*). There was a significant correlation between the heart rate during exercise and the SW velocity after MVC (*r* = 0.78, *P* < 0.001; Supplementary data online, *Figure S1*).

**Figure 3:**
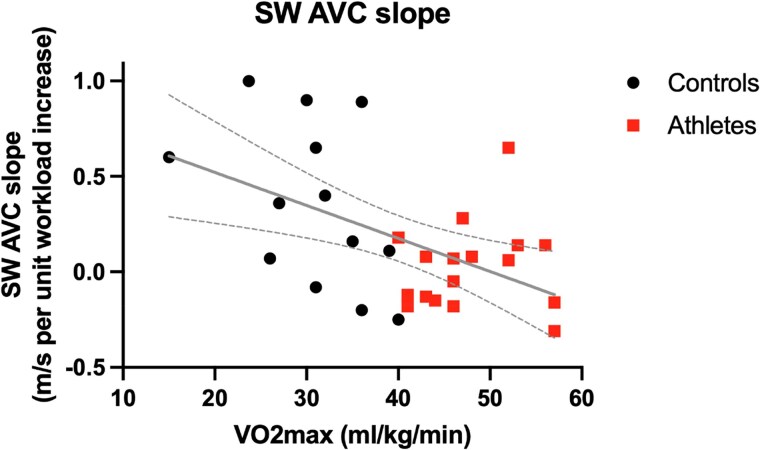
Correlation of SW AVC velocity slopes and VO_2_max. The slope of SW velocity change during exercise after AVC correlated significantly with VO_2_max. The regression line is shown in grey with the dotted lines representing the 95% CI. AVC, aortic valve closure.

The SW velocities after AVC increased significantly in non-athletes, but not in athletes (*Figure 2*). While SW velocity after AVC was equal between the two groups at rest, SW velocity after AVC during peak exercise was 3.6 ± 0.6 m/s in athletes and 4.6 ± 0.8 m/s in non-athletes (*P* = 0.002). The slope of SW velocity after AVC per exercise stage was 0.0 ± 0.8 cm/s in athletes and +1.6 ± 1.6 cm/s in non-athletes (*P* = 0.006). A similar trend was seen for the slope of SW velocity change per heart rate increase (see Supplementary data online, *Figure S2*). There was a significant inverse correlation between the slope of SW increase after AVC and VO_2_max (*r* = −0.51, *P* = 0.003, *Figure 3*).

Sensitivity analyses excluding the one hypertensive participant showed no differences in results (see Supplementary data online, *Table S3*).

Intra- and interobserver reproducibility of the SW velocity after MVC was excellent {ICC 0.97 [95% confidence interval (CI): 0.94–0.99] and 0.89 [95% CI: 0.76–0.95], respectively}, whereas intra- and interobserver reproducibility of the SW velocity after AVC was good [ICC 0.79 (95% CI: 0.59–0.89) and 0.69 (95% CI: 0.40–0.84), respectively].

## Discussion

Invasive studies have previously demonstrated athletes to have reduced chamber stiffness in comparison to sedentary control subjects.^3,21^ In the present study, we confirmed these observations non-invasively using SW elastography. The key findings of this study are that (i) SW velocities after MVC at rest are lower in master athletes than in sedentary non-athletes, and (ii) SW velocities after AVC increase during exercise in non-athletes but remain unchanged during exercise in athletes (*Graphical Abstract*). Our data show that SW elastography reveals differences that are not detected by conventional measurements, suggesting that the non-invasive assessment of myocardial stiffness by SW elastography may push the frontiers of diastolic function assessment in clinical practice.

### Standard diastolic measurements fail to identify athletes’ superior diastolic function

According to current guidelines, the echocardiographic assessment of diastolic function should at least consist of the measurement of septal and lateral e′ velocity, E/e′ ratio, left atrial volume index (LAVI), and TR velocity.^10^ When comparing these and other diastolic parameters between the athletes and the control subjects in this study, we did not find any signs of better diastolic performance in athletes. LAVI was found to be larger in athletes than in non-athletes, which is an established consequence of endurance training.^26^ Diastolic measurements during exercise did not reveal differences in diastolic function between athletes and control subjects.

### SW elastography as a more sensitive tool to depict differences in myocardial stiffness

Several previous studies have demonstrated that SW velocities increase with age.^15,19^ In healthy volunteers aged 40–59 years, SW velocities of 3.8 ± 0.8 were reported both after MVC and AVC.^19^ In volunteers aged 60–80 years, SW velocities were found to be 4.5 ± 1.1 m/s after MVC and 4.3 ± 0.6 m/s after AVC. The higher SW velocities in older subjects reflect the natural process of myocardial stiffening with increasing age.

In the current study, the SW propagation velocity after MVC at rest was found to be lower in athletes than in non-athletes. We presume that this difference can be attributed to the reduced intrinsic myocardial stiffness in athletes.^3,21^ Additionally, variations in filling pressures may contribute to this difference, as demonstrated by a recent SW study.^27^ During exercise, we observed that the SW velocity after MVC increased equally and reached similar values in both groups during exercise. The increase of SW velocity after MVC in athletes and controls during exercise seems to resemble the increase in systolic wall stress as observed in previous exercise studies.^28^ Since MVC is directly followed by the isovolumic contraction phase, it is conceivable that the faster SW propagation velocity after MVC during exercise reflects active stiffness due to an increase in contractility during this phase. These findings are supported by a recent study demonstrating a significant increase in SW velocity after MVC following dobutamine administration in pigs.^27^ A strong relationship was found between the SW velocity after MVC and dP/dV after dobutamine, indicating that SW velocity after MVC is related to operational wall stress under dynamic conditions. Furthermore, our exercise data showed a strong correlation between heart rate and SW velocity after MVC. The higher state of contractility at higher heart rates during exercise presumably leads to faster active stiffening of the myocardium, which is measured as a higher SW velocity at the moment of MVC. SW velocities after MVC seem to reflect passive myocardial stiffness at rest, but as soon as exercise starts and contractility increases, this SW velocity is predominated by active stiffness.

The most interesting findings during exercise were found after AVC. Control subjects showed a significant increase in SW velocity after AVC during exercise. However, in athletes, this SW velocity remained equal to the baseline value during the entire test, even at peak exercise. Since AVC is directly followed by the isovolumic relaxation phase, it is conceivable that the SW velocity after AVC is related to relaxation properties of the myocardium. While exercise in sedentary control subjects leads to a significant increase in active stiffness during this phase due to an increase in contractility, athletes have very fast and efficient relaxation,^29,30^ leading to a fast decay in elastance and consequently lower SW velocities during the isovolumic relaxation phase. The extent of increase in SW velocity after AVC may therefore reflect the early relaxation rate. The difference between athletes and non-athletes that was unmasked by SW elastography could not be detected by conventional parameters such as e′ velocity, which confirms that the conventional diastolic parameters lack sensitivity to detect subtle differences. Interestingly, the slope of SW velocity after AVC was inversely related to VO_2_max, which demonstrates that this slope is related to an individual’s exercise performance. It remains to be investigated whether this parameter could serve as a precursor for diastolic dysfunction in apparently healthy individuals.

### Limitations

This study has several limitations to address. First, the sample size of this study is small. However, the purpose of this study was explorative. Future studies with more participants should be conducted to investigate the heterogeneity in SW response during exercise among individuals and to investigate the clinical potential of these measurements. Second, we measured SW velocity as a surrogate of myocardial stiffness, but we did not have a gold standard for myocardial stiffness. In future studies, it would be interesting to compare the findings with invasive measurements such as −dP/dt and Tau to gain more knowledge on the exact physiology of different SWs during exercise. Last, this study exclusively contained male endurance athletes, but it remains unknown whether our results are generalizable to non-endurance athletes or female athletes.

## Conclusion

SW elastography has the ability to unmask subtle differences in myocardial stiffness between master athletes and sedentary control subjects. SW velocities after MVC are lower at rest in master athletes, confirming reduced myocardial stiffness in athletes. SW velocities after AVC increase in non-athletes during exercise, but not in athletes, presumably reflecting differences in relaxation properties. Future studies should investigate whether this non-invasive measurement can be used as an early marker for diastolic dysfunction.

## Supplementary data

Supplementary data are available at *European Heart Journal - Imaging Methods and Practice* online.

## Consent

All subjects provided written informed consent prior to participation.

## Supplementary Material

qyaf023_Supplementary_Data

## Data Availability

The data underlying this article will be shared on reasonable request to the corresponding author.

## Lead author biography

Dr Karim Taha (MD, PhD) is a cardiology resident at the University Medical Center Utrecht, The Netherlands, with a focus on exercise echocardiography and advanced echocardiographic techniques, including strain imaging and ultra-high frame rate imaging. Supported by a personal EACVI research grant, he completed a research fellowship at University Hospital Leuven, where he conducted the current study.
