# Supplementary material for: Shear wave elastography to unmask differences in myocardial stiffness between athletes and sedentary non-athletes
Source: Eur Heart J Imaging Methods Pract. 2025 Mar 21;2(4):qyaf023. doi: 10.1093/ehjimp/qyaf023 (PMC11925635; doi:10.1093/ehjimp/qyaf023)
Supplement: qyaf023_Supplementary_Data [file qyaf023_supplementary_data.docx]

**Supplementary material**

**Supplementary table 1. Exercise echocardiography protocol**

|  | Rest | 25% | 50% | 75% |
| --- | --- | --- | --- | --- |
| PLAX (HFR) | ● | ● | ● | ● |
| A4C (B-mode) | ● | ● |  | ● |
| A4C (TDI) | ● | ● |  | ● |
| A2C (B-mode) | ● | ● |  | ● |
| MV inflow (PW) | ● | ● |  | ● |
| Septal e’ (TDI PW) | ● | ● |  | ● |
| Lateral e’ (TDI PW) | ● | ● |  | ● |
| LVOT VTI (PW) | ● | ● |  | ● |
| TR velocity w/ agitated colloid (CW) | ● | ● |  | ● |

**Supplementary table 2. Hemodynamic measurements during semi-supine exercise**

|  | Master athletes | Non-athletes | P-value |
| --- | --- | --- | --- |
| **Heart rate (bpm)** |  |  |  |
| Rest | 60 ± 9 | 69 ± 7 | <0.003 |
| 25% | 95 ± 12 | 99 ± 8 | 0.239 |
| 50% | 119 ± 14 | 120 ± 11 | 0.749 |
| 75% | 149 ± 20 | 147 ± 18 | 0.747 |
| **Cardiac output (L/min)** |  |  |  |
| Rest | 5.3 ± 1.1 | 5.5 ± 1.0 | 0.672 |
| 25% | 10.0 ± 1.6 | 9.5 ± 2.3 | 0.458 |
| 75% | 14.5 ± 2.7 | 11.9 ± 2.0 | 0.035 |
| **Systolic BP (mmHg)** |  |  |  |
| Rest | 129 ± 12 | 136 ± 21 | 0.333 |
| 25% | 157 ± 17 | 162 ± 24 | 0.566 |
| 50% | 187 ± 26 | 181 ± 24 | 0.562 |
| 75% | 210 ± 28 | 193 ± 25 | 0.161 |
| **Diastolic BP (mmHg)** |  |  |  |
| Rest | 80 ± 12 | 80 ± 12 | 0.983 |
| 25% | 85 ± 12 | 93 ± 13 | 0.121 |
| 50% | 94 ± 10 | 101 ± 15 | 0.209 |
| 75% | 98 ± 22 | 107 ± 17 | 0.322 |

**Supplementary figure 1: Correlation of SW velocity after MVC and heart rate**

**
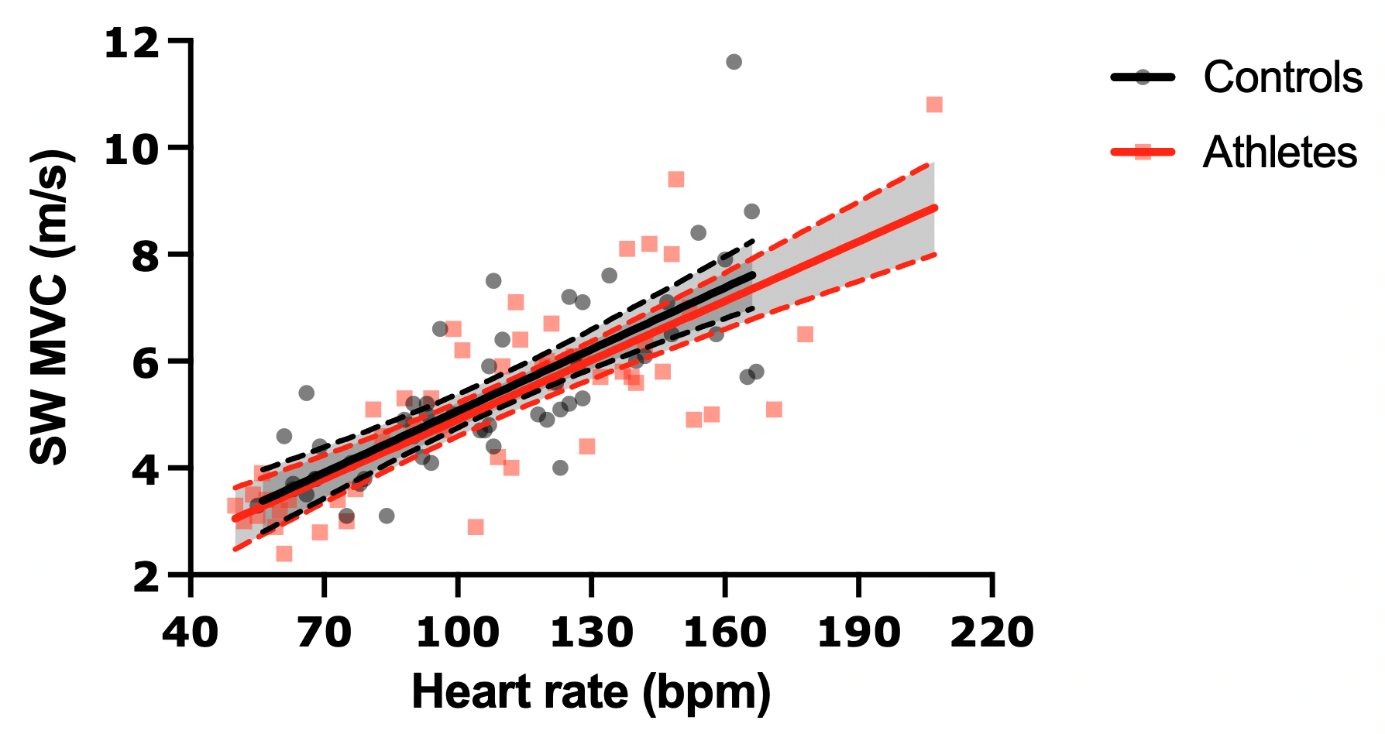
**

There was no significant difference between the correlation slopes of the two groups (p=0.204). SW MVC = shear wave velocity after mitral valve closure.

**Supplementary figure 2: Correlation of SW velocity after AVC with heart rate**


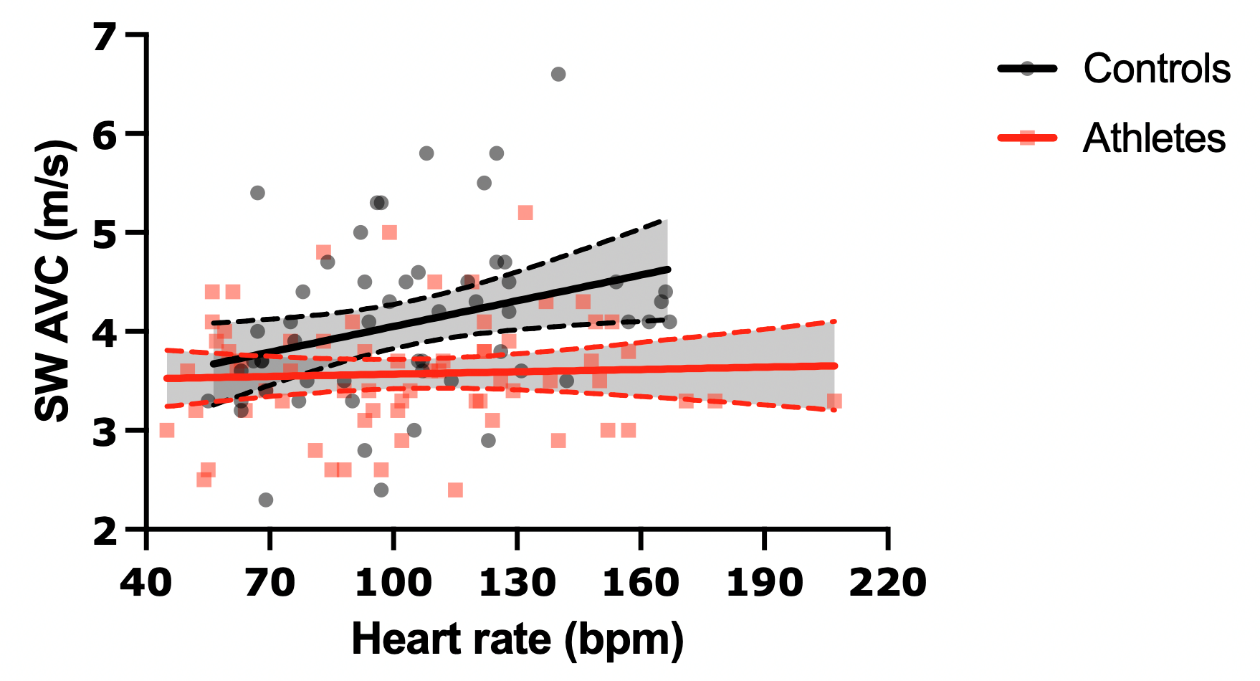


There was a significant difference between the correlation slopes lines of the two groups (p=0.006).

**Supplementary table 3: Sensitivity analysis excluding 1 hypertensive participant**

|  | **Athletes** | **Controls** | **P-value** |
| --- | --- | --- | --- |
| SW MVC rest (m/s) | 3.2 ± 0.4 | 3.7 ± 0.5 | 0.004 |
| SW AVC rest (m/s) | 3.6 ± 0.5 | 3.6 ± 0.5 | 0.876 |
| Slope SW MVC (m/s increase per 25% power output increase) | 1.3 ± 0.5 | 1.2 ± 0.5 | 0.744 |
| Slope SW AVC (m/s increase per 25% power output increase) | 0.0 ± 0.2 | 0.4 ± 0.4 | 0.006 |
